# The gastrin-releasing peptide receptor in oncology: a comprehensive review of recent advances in targeted radionuclide theranostics

**DOI:** 10.3389/fonc.2026.1845936

**Published:** 2026-06-08

**Authors:** YunLong Yang, Jiang Fu, ShengJie Tang, Tao Liu, HaiYang Hu, HaiYang Guo, Long Wen, Yang Yang, ChengKuan Liu, GuiYan Yi, Li Yu, HaiNing Zhou

**Affiliations:** 1 Department of Thoracic Surgery, Suining Central Hospital, Suining, Sichuan, China; 2 Institute of Surgery, Graduate School, Chengdu University of Traditional Chinese Medicine, Chengdu, China; 3 Institute of Surgery, Graduate School, North Sichuan Medical College, Nanchong, China; 4 Department of Thoracic Surgery, Affiliated Hospital of Southwest Medical University, Luzhou, China; 5 College of Medical Technology, Chengdu University of Traditional Chinese Medicine, Chengdu, China; 6 Department of Physical Examination, Suining Central Hospital, Suining, Sichuan, China

**Keywords:** cancer, diagnosis, GRPR, radiopharmaceutical, targeted-therapy

## Abstract

The gastrin-releasing peptide receptor (GRPR) is aberrantly expressed in several malignancies, including prostate cancer, breast cancer, lung cancer, and gastrointestinal stromal tumors, making it an important target for molecular imaging and targeted radionuclide therapy. In recent years, advances in radioligand design, radionuclide selection, and theranostic concepts have substantially promoted the development of GRPR-mediated oncologic applications in nuclear medicine. This review systematically summarizes recent progress in GRPR-targeted radioligands for tumor imaging and radioligand therapy, with particular emphasis on their biological rationale, representative tracers, clinical utility, and major limitations across different tumor types. Overall, GRPR-targeted imaging has demonstrated promising diagnostic value in prostate cancer, estrogen receptor-positive breast cancer, and selected gastrointestinal stromal tumors, and may complement existing imaging modalities. From a therapeutic perspective, the development of high-stability antagonists, metabolic protection strategies, and novel therapeutic radionuclides is driving GRPR-targeted radioligand therapy toward improved therapeutic index and greater translational potential. Nevertheless, several challenges remain, including receptor heterogeneity, physiologic uptake in normal tissues, optimization of pharmacokinetics, the complexity of dual-targeted constructs, and the lack of high-quality prospective clinical evidence. Future progress will likely depend on ligand engineering, dual-target strategies, individualized dosimetry, and the integration of imaging-based treatment assessment into a closed-loop precision theranostic framework.

## 1 Introduction

Cancer remains a major challenge to global health. According to the latest estimates, there were nearly 20 million new cancer cases and approximately 9.7 million cancer-related deaths worldwide in 2022, and the global disease burden is projected to rise significantly by 2050 (1, 2). Despite significant advances in surgery, radiotherapy, chemotherapy, targeted therapy, and immunotherapy, cancer-related mortality remains high, reflecting persistent limitations in early diagnosis, accurate risk stratification, and effective treatment of biologically heterogeneous diseases (1, 2). Against this backdrop, receptor-based targeting strategies have attracted increasing attention, as they can selectively recognize tumor-associated cell surface biomarkers, thereby supporting precise imaging and targeted therapeutic delivery.

The gastrin-releasing peptide receptor (GRPR), also known as bombesin receptor subtype 2 (BB2), belongs to the bombesin receptor family and the class A G protein-coupled receptor (GPCR) superfamily (3–5). Under physiological conditions, GRPR is involved in regulating gastrointestinal motility, gastric secretion, smooth muscle contraction, and pancreatic enzyme release (6). However, abnormal GRPR activation is also considered to be involved in tumor biology by regulating a variety of downstream signaling pathways, including ERK, JNK, p38, NF-κB, and Rho-related cascades, thereby promoting proliferation, survival, angiogenesis, invasion, and metastatic potential (7, 8).

Importantly, GRPR is overexpressed in a variety of solid malignant tumors, including prostate cancer, breast cancer, lung cancer, and gastrointestinal stromal tumors (GISTs) (9). This expression profile makes GRPR an attractive molecular target in nuclear oncology. Unlike traditional anatomical imaging modalities such as CT and MRI, GRPR-targeted imaging can provide biologically relevant information at the receptor level and may improve the detection of receptor-positive lesions, especially in the context of molecular heterogeneity. Meanwhile, the development of GRPR-directed radioligand therapy (RLT) has expanded the clinical significance of this target beyond mere diagnosis, supporting a therapeutic paradigm where the same molecular axis can be used for lesion visualization, patient selection, dosimetry, and treatment.

Based on this, GRPR-mediated theranostic strategies have become a research hotspot in nuclear oncology and precision cancer therapy. This review aims to systematically summarize the latest research advances of GRPR-targeted radioligands in tumor imaging and RLT, with a focus on summarizing their application basis, clinical potential, and main limitations in different tumor types, and further discuss the key issues including ligand design, radionuclide selection, receptor heterogeneity, and clinical translation. Through systematic collation of existing evidence, this review intends to provide a theoretical basis and research ideas for the subsequent development of high-efficiency GRPR-targeted radiopharmaceuticals and their applications in precision medicine.

Although the present review focuses on GRPR-targeted applications in oncology, GRPR is not exclusively relevant to malignant disease. Previous studies have shown that GRP/GRPR signaling contributes to gastrointestinal development and tissue homeostasis, including villus formation in the gastrointestinal tract (10). In addition, GRPR has been identified as a key mediator of itch transmission in the spinal cord, highlighting its functional importance in the nervous system (11). More broadly, recent evidence suggests that dysregulated GRP/GRPR signaling may also be involved in several non-oncological conditions, including inflammatory, fibrotic, cardiovascular, and neurologic disorders (7). Therefore, a broader understanding of GRPR biology may not only deepen our knowledge of its pathophysiological significance, but also provide useful insights for ligand design, safety evaluation, and the future expansion of GRPR-targeted applications beyond oncology.

## 2 Expression and diagnosis of GRPR in some tumors

Against this broader biological background, the oncologic overexpression of GRPR has attracted particular interest because it enables receptor-targeted molecular imaging and radionuclide theranostics across several tumor types. To facilitate a comprehensive understanding of the research positioning and clinical significance of GRPR-targeted imaging in different cancer types, (**Table 1**) summarizes the most representative application scenarios, advantages, and limitations to date. In the following sections, we will discuss the research progress separately in prostate cancer, breast cancer, lung cancer, and GISTs.

**Table 1:** Representative applications and clinical implications of GRPR-targeted imaging across different tumor types.

Tumor type	GRPR expression pattern and biological relevance	Representative tracers/approaches	Main clinical utility	Potential advantage over current imaging	Key limitations and current role
Prostate cancer	GRPR is overexpressed in a subset of prostate cancers and is partly complementary to PSMA; expression may vary with androgen signaling and ADT exposure	^68^Ga-RM2; GRPR/PSMA dual-tracer or dual-target strategies	Primary tumor detection, lesion localization, complementary molecular stratification, and support for biopsy decision-making	Provides receptor-level information and may complement PSMA in selected settings	Uptake in benign prostate tissue and hyperplasia may reduce specificity; currently best positioned as a complementary imaging tool rather than a replacement for PSMA.
Breast cancer	GRPR expression is particularly relevant in ER-positive/HER2-negative disease and may be useful in low-glycolytic or biologically heterogeneous lesions	GRPR PET; complementary use with FDG, FES, or HER2-directed imaging	Lesion detection, subtype-oriented characterization, whole-body receptor mapping, and potential patient selection	May overcome some limitations of FDG in luminal tumors and may be valuable in invasive lobular carcinoma or receptor-heterogeneous disease	Evidence is still limited, with relatively small cohorts; physiological uptake may be influenced by hormonal status; currently most suitable as a supplementary modality in selected molecular subgroups.
Lung cancer	Lung cancer is biologically heterogeneous, and GRPR may capture specific tumor phenotypes not covered by single-target imaging	^99m^Tc-RGD-BBN and other GRPR/integrin αvβ3 dual-target probes; GRPR-based nanotheranostic platforms	Tumor-versus-inflammation discrimination, detection of primary and metastatic lesions, and characterization of heterogeneous disease	May reduce inflammation-related false positives compared with ^18^F-FDG; dual-target design may improve lesion coverage and specificity	Current evidence is predominantly preclinical; translational relevance remains to be established; currently more appropriate as a scenario-specific adjunct to FDG imaging.
Gastrointestinal stromal tumor (GIST)	A subset of GISTs shows imageable GRPR expression; wild-type and SDH-deficient GISTs may represent promising indications	RM26, NeoB, and related theranostic-pairing strategies	Receptor-specific imaging, patient stratification, and selection for GRPR-targeted RLT	May offer an additional stratification approach in patients with limited standard therapeutic options and may facilitate imaging-to-therapy linkage	Available studies are small and heterogeneous; GRPR PET reflects target expression rather than total tumor burden and cannot replace CT or MRI; current value lies mainly in identifying candidates for GRPR-targeted therapy.
Glioma	GRPR expression has been demonstrated in human gliomas, supporting the biologic rationale for receptor-targeted imaging in brain tumors	^68^Ga-NOTA-Aca-BBN (7–14); exploratory antagonist-based ^177^Lu-labeled GRPR ligands	Tumor detection/localization, adjunct to surgical planning, and potential future theranostic stratification.	High tumor-to-background contrast relative to normal brain tissue; may complement MRI in lesion characterization	Therapeutic translation is limited by the blood-brain barrier and infiltrative tumor margins; current role is mainly exploratory imaging, with theranostic application still at a preclinical or early translational stage.
Ovarian cancer	GRPR expression and specific bombesin/GRP binding have been identified in a subset of ovarian cancers, indicating a potentially targetable receptor population	Bombesin/GRPR-targeted probes; bombesin-based targeted therapeuticlogs in preclinical models	Currently exploratory, with possible future use in biomarker-based patient selection for GRPR-targeted imaging or therapy	May provide a receptor-based precision strategy in selected patients beyond morphology-based assessment. This is still a potential rather than an established clinical advantage	Evidence remains predominantly preclinical, and dedicated clinical GRPR theranostic data are still lacking; current role is therefore hypothesis-generating rather than practice-changing.

### 2.1 Prostate cancer

Although transrectal ultrasound-guided prostate biopsy (TRUS-biopsy) remains the traditional gold standard for the diagnosis of prostate cancer and plays an important role in tumor detection, lesion localization, invasiveness assessment, and treatment decision-making (12), its “blind sampling” characteristic limits its coverage of tumor spatial heterogeneity. A study conducted by Ahmed et al. showed that TRUS biopsy carries a high risk of missed diagnosis and misstratification for clinically significant prostate cancer (csPCa), with a misdiagnosis or misclassification rate of 18% for csPCa and 32% for non-clinically significant prostate cancer (ncsPCa); 44 severe complications were also reported, including 8 cases of sepsis (13). This result suggests that although TRUS biopsy still holds an important clinical position, it has obvious limitations in both diagnostic accuracy and safety.

In contrast, multiparametric magnetic resonance imaging (mpMRI) has high sensitivity for csPCa and helps reduce unnecessary biopsies (14, 15); however, mpMRI remains insufficient in identifying small and low-volume lesions within the prostate, and its diagnostic efficacy is highly dependent on image acquisition quality and reader experience. Inter-reader variability can significantly increase the missed diagnosis or misdiagnosis rate of csPCa, which has been reported to be as high as 35% in some studies (14, 15). In addition, although ^68^Ga-PSMA-11 PET/CT and PET/MRI targeting PSMA are generally superior to mpMRI in overall sensitivity and specificity, their ability to detect low-grade tumors and some heterogeneous lesions is still relatively limited, resulting in a certain risk of false negatives in practical applications.

Overall, a single diagnostic modality is difficult to fully cover the molecular and biological heterogeneity of prostate cancer, thus there is an urgent need for new molecular imaging targets as supplements (16).

Against this backdrop, GRPR-targeted molecular imaging has gradually emerged as a research hotspot and is believed to have the potential to compensate for the limitations of PSMA-targeted imaging in certain clinical scenarios, particularly for the identification of lesions with low PSMA expression, obvious heterogeneity, or those associated with PSMA-targeted therapy resistance (16). Rinne et al. systematically investigated RM26, a GRPR antagonist suitable for SPECT, PET imaging, and targeted therapy. The results showed that RM26 variants labeled with ^68^Ga, ^55/57^Co, and ^111^In all exhibited high receptor affinity, achieving significant uptake in GRPR-positive tumors, while showing low accumulation in GRPR-negative organs and rapid clearance from the circulation (17–20). These findings indicate that RM26 possesses a favorable pharmacokinetic basis as a GRPR molecular probe, laying an important foundation for subsequent probe optimization.

Furthermore, the modified construct [^66^Ga]Ga-NOTA-PEG_2-RM26 based on RM26 successfully achieved clear visualization of GRPR-positive xenografts in microPET/MR, suggesting that optimization of linkers and chelation systems can further improve the imaging contrast of local prostate lesions (18–20). The important significance of this research direction lies in its verification of the feasibility of GRPR antagonists for prostate cancer imaging at the molecular design level. However, its limitations are also clear: most current evidence is mainly derived from cellular and xenograft models, and there is a lack of unified standards for imaging time windows, model construction, and quantitative indicators among different studies. In the future, larger-sample translational studies should be promoted on the basis of a unified evaluation system to enhance the comparability and clinical interpretability of the results.

In terms of chelator optimization, Dar, as a novel bifunctional chelator (BFC) with abundant donor sites and tunable spatial structure, can stably coordinate with various radioactive metals (e.g., ^68^Ga, ^177^Lu, ^89^Zr), providing higher flexibility for the theranostic development of GRPR probes (21). Luo et al. first introduced Dar-P2 and Dar-C5-P2 into the RM26 peptide chain, synthesizing [^68^Ga]Ga-Dar-C5-P2-RM26 and [^68^Ga]Ga-Dar-P2-RM26. PET/CT imaging in PC-3 xenograft mice revealed that these two probes exhibited superior tumor-specific uptake compared with [^68^Ga]Ga-DOTA-P2-RM26 (22). This study indicates that the imaging performance of GRPR probes depends not only on the ligand itself, but also on the chelator structure, spacer design, and overall molecular configuration.

However, its limitations are evident: current research remains in the preclinical validation stage, and studies on metabolic pathways, long-term *in vivo* retention, safety, and clinical scale-up preparation are still insufficient. In the future, head-to-head human studies and more systematic dosimetry evaluations can be conducted to further clarify the real clinical benefit of Dar-based chelators compared with traditional DOTA/NOTA systems.

In addition to RM26, probes based on the GRPR antagonist RM2 also exhibit strong clinical translational potential. Existing studies have reported that ^68^Ga-RM2 PET/CT has a positive rate of up to 98% in patients with newly diagnosed prostate cancer, indicating high sensitivity in lesion detection (23). Rosalba Mansi et al. labeled RM2 with ^111^In and ^68^Ga respectively, and conducted *in vivo* distribution and PET imaging studies in PC-3 and LNCaP xenograft mouse models. The results showed that both probes exhibited significant tumor-specific uptake, along with high GRPR affinity and selectivity (24). These studies collectively indicate that RM2-based probes possess a favorable imaging biological basis and clinical translational prospects.

It should be noted that a high positive rate does not equate to high specificity; due to the expression of GRPR in some benign prostate tissues and benign prostatic hyperplasia (BPH) tissues, sole reliance on RM2 or other GRPR probes for diagnosis may pose a certain risk of false positives (25, 26). Therefore, the authors believe that RM2 is more suitable as a supplementary tool for PSMA or mpMRI, rather than an independent method to completely replace existing imaging modalities at this stage.

The application of GRPR-targeted imaging in biochemical recurrence (BCR) after prostate cancer treatment also deserves particular attention. Because conventional imaging modalities, including CT, MRI, and bone scintigraphy, often show limited sensitivity in BCR patients with low PSA levels or low tumor burden, more effective molecular imaging tools are urgently needed. In this context, Duan et al. (27)conducted a prospective phase II head-to-head PET/MRI study comparing with in 27 patients with BCR who had negative or equivocal findings on conventional imaging. The results demonstrated that outperformed in sensitivity (85.7% *vs*. 71.4%), accuracy (88.9% *vs*. 77.8%), and negative predictive value (83.3% *vs*. 50.0%). Moreover, identified 14 additional lesions missed by in 6 patients and showed higher lesion uptake intensity, suggesting that GRPR-targeted imaging may provide incremental diagnostic value in selected BCR settings (27).

The significance of this study lies in its direct demonstration that a GRPR-targeted tracer may outperform a PSMA-targeted tracer in specific clinical scenarios of BCR, thereby supporting the potential role of GRPR imaging as a complementary molecular approach in recurrent prostate cancer. Preliminary findings from the first interim analysis of the same prospective study also suggested the feasibility and clinical promise of head-to-head comparison between and PET/MRI in this setting (28). Nevertheless, these results should still be interpreted with caution because of the relatively small sample size, substantial inter-patient heterogeneity, and the lack of uniform pathological confirmation for all detected lesions. Therefore, larger prospective multicenter studies are still required to validate these observations. Future investigations should further stratify patients according to PSA level, previous treatment history, hormone status, and lesion type, in order to better define the subgroups most likely to benefit from imaging.

In addition to ligand structure, radionuclide selection is also a crucial factor affecting the clinical application of GRPR probes. Although ^68^Ga is a commonly used PET radionuclide, its short half-life (approximately 68 minutes) imposes certain limitations on probe preparation, transportation, and delayed imaging. In contrast, ^44^Sc possesses a longer half-life (approximately 4 hours) and favorable imaging physical properties, making it a promising candidate to improve the adaptability of probe administration and imaging time windows. Kálmán-Szabó et al. compared two probes, [^68^Ga]Ga-NODAGA-AMBA and [^44^Sc]Sc-NODAGA-AMBA, based on the bombesin (BBN) analog AMBA. The results showed that both probes could achieve specific GRPR binding and tumor visualization, while [^44^Sc]Sc-NODAGA-AMBA exhibited superior *in vitro* stability, with tumor-targeting ability comparable to that of the ^68^Ga-labeled probe and lower background signal (29). This study indicates that the optimization of GRPR imaging can be achieved not only through ligand modification, but also through radionuclide replacement, thereby improving pharmacokinetic properties and clinical workflow adaptability.

However, its limitations are notable: current research is still dominated by animal experiments, and the production supply, cost, dosimetric benefits, and clinical accessibility of ^44^Sc remain to be further verified. In the future, if a more mature radionuclide preparation and distribution system can be established, ^44^Sc-based GRPR probes are expected to demonstrate greater advantages in delayed imaging and multi-center applications.

Given the significant heterogeneity in the expression of PSMA and GRPR in prostate cancer, bispecific heterodimers have emerged as a focus of research in recent years. Liolios et al. designed and synthesized a novel bispecific heterodimer compound 3, which covalently links the PSMA ligand PSMA-617 and the GRPR antagonist RM2 via DOTA. *In vitro* studies demonstrated that this probe maintained receptor affinity comparable to that of monomers, while exhibiting superior cellular binding and internalization. Molecular docking simulations further confirmed its ability to simultaneously recognize both PSMA and GRPR.

*In vivo* distribution experiments showed that [^68^Ga]Ga-3 could simultaneously target PSMA-positive and GRPR-positive tumors, and compared with monomers, it exhibited higher tumor uptake, faster blood clearance, and lower renal uptake (30). Mitran et al. constructed a short-peptide heterodimer NOTA-DUPA-RM26 based on DUPA and RM26, and completed labeling with ^111^In and ^68^Ga. The results indicated that although its *in vitro* affinity for a single target was slightly lower than that of monomers, it still achieved dual-specific targeting *in vivo*, along with rapid blood clearance, favorable tumor uptake, and extremely low renal accumulation (31).

Collectively, these studies demonstrate that bispecific heterodimers hold significant potential in addressing the molecular heterogeneity of prostate cancer and may provide a new approach for the development of safer theranostic agents. However, such probes still face challenges including complex structure, high difficulty in synthesis and scale-up, narrow window for pharmacokinetic optimization, and insufficient evidence of clinical safety. In the future, systematic optimization can be conducted focusing on linker length, charge distribution, hydrophilic-hydrophobic balance, and radionuclide compatibility, and combined with standardized dosimetric and toxicological studies to promote clinical translation.

It is important to emphasize that while GRPR-targeted imaging holds broad prospects, its limitations cannot be overlooked. First, GRPR expression is regulated by the androgen axis, and androgen deprivation therapy (ADT) can significantly alter the expression level of GRPR in prostate cancer tissues (32), thereby affecting the stability and interpretability of imaging results. Second, GRPR is not exclusive to malignant tissues; it is also expressed in benign prostate tissues and BPH, which may compromise its specificity and increase the risk of false positives (25).

Head-to-head clinical studies have further demonstrated that ^68^Ga-RM2 and ^68^Ga-PSMA-11 PET exhibit comparable sensitivity in prostate cancer detection, both of which are higher than that of mpMRI; however, in terms of specificity, mpMRI is the highest, followed by ^68^Ga-PSMA-11, with ^68^Ga-RM2 slightly lower (26). This result indicates that GRPR imaging is more adept at “detecting lesions” but requires cautious interpretation in “distinguishing malignant from benign lesions”.

For this reason, a more reasonable positioning of GRPR is as a supplement to PSMA rather than a replacement. Existing studies have shown that GRPR and PSMA expression are to a certain extent independent of each other (33); the dual-tracer or dual-target strategy can improve the overall detection rate and reduce unnecessary biopsies without increasing the missed diagnosis rate of csPCa. Relevant clinical data have shown that integrating GRPR and PSMA dual-tracer PET/CT can significantly reduce the proportion of unnecessary biopsies (by approximately 52.7%) and achieve a higher dual-tracer detection rate (approximately 77.4%) (34).

Therefore, the authors believe that the most critical issue in the next stage of GRPR research is no longer “whether imaging is feasible”, but rather “how to standardize image interpretation and integrate GRPR-targeted PET into clinical decision-making pathways”. In this regard, a concrete step has already been proposed. Duan et al. introduced the modified PROMISE criteria (mPROMISE) for GRPR-targeted PET, adapted from the Prostate Cancer Molecular Imaging Standardized Evaluation framework originally developed for PSMA-ligand PET (23). This structured reporting system incorporates assessment of GRPR expression together with disease localization in the prostate bed (T), lymph nodes (N), skeleton (Mb), and other organs (Mc), thereby providing a standardized framework for scan interpretation and reporting. Importantly, in a retrospective evaluation of prospectively acquired PET datasets from patients undergoing initial staging and biochemical recurrence assessment, mPROMISE demonstrated substantial to almost perfect interreader agreement across the major interpretive categories, supporting its feasibility and reproducibility in GRPR-targeted imaging (23). Accordingly, future multicenter studies should not only optimize probe performance, but also actively incorporate structured interpretation systems such as mPROMISE to improve reporting consistency, cross-study comparability, and clinical utility.

Overall, existing evidence demonstrates that GRPR-targeted molecular imaging has clear complementary value in the initial diagnosis of prostate cancer, detection of recurrent lesions, and identification of PSMA-low-expression lesions (16, 23, 27, 33, 34). Studies on RM26, RM2, NeoB, and PSMA/GRPR bispecific heterodimers collectively indicate that the greatest clinical significance of GRPR lies not in replacing PSMA, but in complementing PSMA, mpMRI, and pathological information, thereby more comprehensively covering the molecular heterogeneity of prostate cancer (26, 33, 34).

Future research should focus on three key areas: first, conducting large-sample, multi-center, prospective studies with adequate pathological control to clarify the optimal indications of different GRPR probes in various clinical scenarios; second, promoting the unification of imaging acquisition parameters, quantitative analysis methods, and interpretation standards to improve the cross-center reproducibility of results; third, systematically integrating GRPR imaging with PSMA, mpMRI, and clinicopathological parameters to establish a combined diagnostic model based on molecular phenotypes. Only with the synchronous advancement of “probe optimization, clinical validation, and standardized application”, can GRPR-targeted imaging be truly transformed from a promising research tool into a routine component in the precise diagnosis and management of prostate cancer.

### 2.2 Breast cancer

Mammography remains one of the most mature and widely used techniques in current breast cancer screening; however, its diagnostic efficacy is significantly influenced by breast density, tissue background, and individual factors. Previous studies and large-scale screening data have shown that mammography has a certain proportion of false-positive results, which often lead to additional imaging examinations, short-term follow-up, or even unnecessary core needle biopsies, thereby increasing the risk of psychological burden on patients, increased medical costs, and decreased compliance with subsequent screening (35–37). This issue is particularly prominent in breasts with abundant fibroglandular components or obvious image overlap.

For women with dense breasts, breast implants, and some receiving hormone replacement therapy, the sensitivity of mammography is further reduced, primarily due to the “masking effect” of high-density fibroglandular tissue on tumors. Therefore, although mammography is suitable as a basic screening tool, its negative results in specific populations cannot fully rule out the presence of tumors, which also constitutes an important clinical context for the development of complementary molecular imaging techniques (38).

With excellent soft tissue resolution and dynamic contrast enhancement capabilities, breast MRI is generally superior to mammography and ultrasound in breast cancer detection. Previous meta-analyses have shown that the overall sensitivity of breast MRI for suspicious breast lesions is approximately 90%, while its specificity is about 72%, indicating its fundamental characteristic of “high sensitivity but relatively low specificity” (39). This indicates that breast MRI has unique advantages in detecting additional lesions, assessing lesion extent, and supplementary screening for dense breasts; however, it also tends to generate more suspicious enhancing lesions and false-positive results. Furthermore, practical factors such as high cost, long examination time, strict requirements for equipment and reader experience, and inapplicability to all patients mean that breast MRI remains unable to independently undertake the task of universal precise stratification.

**Figure 1:**
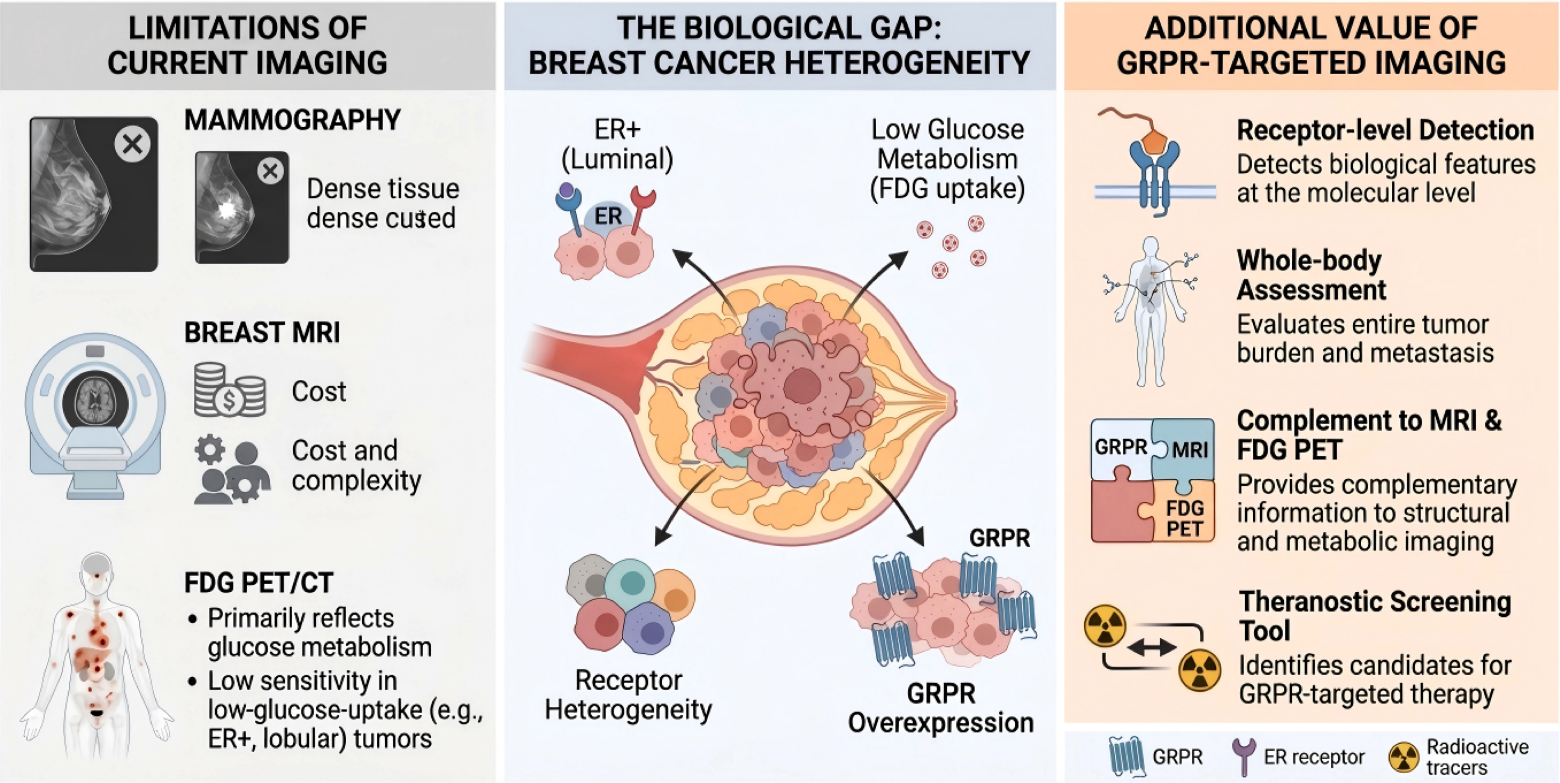
Rationale for GRPR-targeted imaging as a complementary approach in breast cancer. Current imaging modalities in breast cancer remain limited by structural and metabolic constraints, including reduced performance of mammography in dense breasts, the high cost and complexity of MRI, and the low sensitivity of ^18^F-FDG PET/CT in tumors with low glucose metabolism. Given the biological heterogeneity of breast cancer, especially in ER-positive/luminal phenotypes with potential GRPR overexpression, GRPR-targeted imaging may fill this diagnostic gap by providing receptor-based whole-body assessment, complementary information to MRI and FDG PET, and a screening tool for GRPR-targeted theranostic approaches.

In other words, the core challenge currently facing breast cancer imaging is not “whether more lesions can be detected”, but “how to maintain acceptable specificity and accessibility while improving sensitivity”. This also explains why targeted molecular imaging, particularly receptor imaging that can reflect tumor biological characteristics, is gradually emerging as an important complementary direction for precision diagnosis of breast cancer (39, 40) (**Figure 1**).

Among various candidate molecular targets, GRPR has attracted widespread attention because it is frequently expressed in breast cancer, particularly in estrogen receptor (ER)-positive/luminal subtypes. A large immunohistochemical study of 1, 432 primary breast cancers by Morgat et al. (41)showed that GRPR expression was closely associated with favorable biological features, especially ER positivity, supporting its relevance as a molecular target in luminal breast cancer. In addition, Dalm et al. (42)reported that high GRPR mRNA expression was significantly associated with ESR1-positive tumors and that GRPR expression was largely preserved between primary tumors and paired metastases, suggesting potential applicability in both primary and metastatic settings. This biological association has also been supported by *in vivo* imaging studies. Stoykow et al. (43)found that all PET-positive primary breast tumors on PET/CT were ER- and PR-positive, and that ER expression was the only independent predictor of tracer uptake. Similarly, Zang et al. (44) showed that positivity on PET/CT and tumor uptake intensity were significantly associated with ER expression. Morgat et al. further conducted comparative tissue microimaging on 14 breast cancer samples (10 primary tumors and 4 associated lymph node metastases), showing discordant uptake patterns between and: in ER-positive and low Ki-67 tumors, binding was significantly higher than uptake, whereas in ER-negative and high Ki-67 tumors, uptake was more prominent.

From a translational perspective, the key value of this research is not to prove that GRPR is superior to FDG, but to demonstrate that GRPR imaging can reflect different tumor biological characteristics complementary to FDG metabolism, thereby providing a new approach for stratified diagnosis of breast cancer subtypes. It should be noted that this study had a small sample size and was mainly based on ex vivo samples and limited preoperative imaging comparisons, which is insufficient to directly define its clinical indications. In addition, existing reviews have shown that the high expression rate of GRPR in breast cancer ranges from 62% to 96%, and it is more commonly observed in ER-positive tumors and associated lymph node/distant metastases (43). This “high expression but not universally homogeneous expression” feature suggests that GRPR is suitable as a stratified imaging target rather than a universal marker for all breast cancer subtypes.

Regarding preclinical probe development, Baun et al. (45)evaluated the imaging potential of ^55^Co-DOTA-RM26 in ER-positive breast cancer models, using ^57^Co-DOTA-RM26 as a surrogate tracer for *in vitro* studies. Results demonstrated that this probe could achieve specific targeting in T47D cells and xenograft mice; *in vivo* studies showed that the tumor-to-background ratio (TBR) improved over time, with clearer tumor visualization on PET imaging at later time points, suggesting that ^55^Co with a longer half-life facilitates delayed imaging and enhanced contrast (45). Compared with ^68^Ga-NOTA-RM26 reported by Varasteh et al. (46), the ^55^Co-labeled probe exhibited more favorable time windows and imaging performance in similar models, highlighting that radionuclide selection affects not only “whether imaging is feasible” but also “the optimal timing for imaging”.

The significance of such studies lies in providing a radiochemical basis for subsequent theranostic development; however, their limitations are equally prominent: current evidence is still derived from mouse models, and the density of GRPR expression varies significantly among different models, requiring caution when extrapolating results to humans. In the future, it is more worthwhile to promote head-to-head comparisons based on unified models and standardized peptide doses, and link imaging endpoints to actual treatment-related endpoints, rather than merely focusing on comparisons of percentage injected activity per gram (%IA/g) or standardized uptake value (SUV).

In terms of clinical translation, Wong et al. (47)reported the first prospective human study of in patients with ER/PR-positive, HER2-negative metastatic breast cancer. This study enrolled 7 patients who completed all scheduled examinations, including PET/CT, PET/CT, diagnostic CT, and bone scintigraphy within 3 weeks. The tracer was generally well tolerated, with no severe adverse events observed, and dosimetric analysis showed a whole-body effective dose of approximately 1.9 mSv/200 MBq, supporting favorable clinical feasibility. From a diagnostic perspective, identifiable metastatic lesions were detected in 5 of 7 patients. Notably, in the invasive lobular carcinoma subtype, uptake was higher than that of, and one case of extensive metastatic disease missed by was successfully detected by (47). This first-in-human result is biologically plausible because GRPR is highly expressed in ER-positive/luminal breast cancer: Morgat et al. (48)demonstrated GRPR overexpression in 75.8% of 1, 432 primary breast cancers, with the strongest association seen in ER-positive tumors, and GRPR expression was also retained in most metastatic lymph nodes. In addition, Stoykow et al. (43) showed that all PET-positive primary breast tumors were ER- and PR-positive, whereas Michalski et al. (49) further reported that strong RM2 binding was preserved in all metastatic lesions in 6 of 8 patients with pre-treated ER-positive metastatic breast cancer, supporting the persistence of GRPR targeting potential in advanced disease.

The significance of this study lies in being the first to validate the clinical potential of GRPR-targeted PET in a selected human breast cancer population, especially in luminal/lobular tumors with relatively low FDG avidity. This point is also supported by broader molecular imaging evidence showing that invasive lobular carcinoma is often less conspicuous on PET than ductal carcinoma, highlighting the need for alternative biologically driven tracers. In addition, Wong et al. proposed the possibility of theranostic pairing using, and this concept is supported by preclinical work showing that has favorable stability, significant tumor-growth inhibition, and prolonged survival in GRPR-expressing tumor models. However, the limitations of the breast cancer study remain substantial: the sample size was extremely small, patient heterogeneity was considerable, GRPR IHC findings were not fully concordant with imaging, and the trial was primarily designed to assess feasibility rather than diagnostic efficacy. Therefore, future studies should prioritize larger ER-positive/HER2-negative metastatic cohorts to validate whether the apparent advantages of in invasive lobular carcinoma, bone-dominant disease, and FDG-negative lesions are reproducible, while also clarifying how imaging findings correlate with GRPR expression and whether copper-based theranostics can be translated beyond proof-of-concept (47, 50, 51).

In terms of the theranostic radionuclide system, ^47^Sc has attracted increasing attention in recent years. With a half-life of approximately 3.35 days and emission of 159 keV γ-rays, it possesses both therapeutic potential and SPECT companion imaging capability, thus being regarded as an attractive theranostic nuclide (52–55). Kumar et al. (56)constructed a GRPR-targeted theranostic pair, ^44^Sc/^47^Sc-LF1, and evaluated its biological distribution and imaging performance in T47D breast cancer xenograft mice. Although the GRPR expression level in T47D cells is lower than that in the classic PC-3 model, [^47^Sc]Sc-LF1 still achieved clear and specific tumor uptake, reaching approximately 6.1%IA/g at 4 hours post-administration, and enabled high-contrast SPECT/CT imaging. This indicates that the probe is not dependent on extremely high GRPR expression to exert its imaging value and may also be effective for tumors with moderate GRPR expression.

More importantly, the study suggested a potential synergistic effect when this probe was combined with everolimus, indicating that GRPR-targeted radiotherapy may be optimized by combination with molecular targeted drugs in the future (56). However, current research in this field is still dominated by preclinical validation, and there remains a paucity of real therapeutic data in breast cancer models. In future studies, it is necessary to further clarify the dosimetric advantages, tissue toxicity profiles, and optimal administration regimens of ^47^Sc compared with other therapeutic radionuclides such as ^177^Lu, and avoid overestimating its actual translation speed merely based on its “radionuclide novelty”.

Given the significant molecular heterogeneity of breast cancer, reliance on a single receptor target is often insufficient to cover all lesions, making bispecific heterodimers targeting dual molecular targets an important research direction in recent years (57–63). Mechanistically, the GRPR and integrin αvβ3 reflect distinct biological processes of breast cancer: GRPR is closely associated with tumor cell proliferation and differentiation, while integrin αvβ3 is involved in tumor angiogenesis, invasion, and metastatic progression. Integrating these two targets into a single probe is expected to expand lesion coverage and reduce false-negative rates, especially in lesions with heterogeneous receptor expression.

Zhang et al. (64)conducted a prospective study involving 22 breast cancer patients, 11 of whom underwent a head-to-head comparison between ^68^Ga-BBN-RGD (a bispecific probe targeting both GRPR and integrin αvβ3) and ^68^Ga-BBN (a single-target GRPR probe). The results showed that ^68^Ga-BBN-RGD achieved specific visualization of both primary tumors and various metastatic lesions (including lymph node, bone, and lung metastases), with significantly higher maximum standardized uptake value (SUVmax) in primary tumors and bone metastases compared to ^68^Ga-BBN. Notably, ^68^Ga-BBN-RGD detected more lesions overall, including 3 additional primary lesions, 5 more lymph node metastases, 4 more bone metastases, and 2 more lung metastases that were missed by the single-target probe (64).

**Figure 2:**
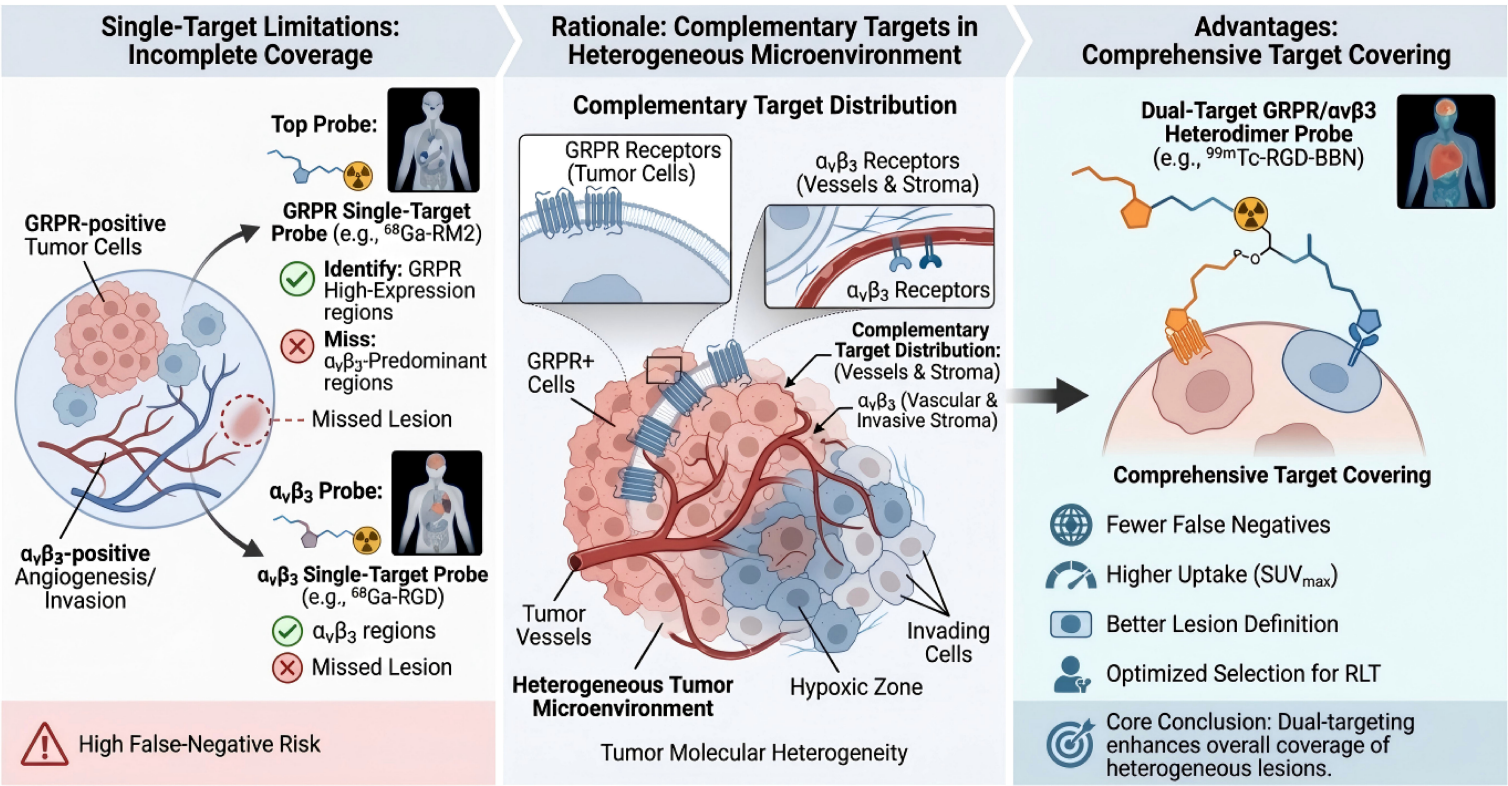
Schematic diagram illustrating the advantages of dual-target heterodimeric probes in molecular imaging of breast cancer. Compared with single-target probes, heterodimeric probes targeting GRPR/αvβ3 can simultaneously cover tumor cell receptor expression and processes associated with tumor angiogenesis and invasion, thereby improving the detection rate of primary lesions and metastases, and particularly helping to reduce false-negative results caused by receptor heterogeneity.

These findings collectively indicate that the dual-target strategy offers not only higher uptake intensity but also broader lesion coverage, particularly in lesions with heterogeneous receptor expression—highlighting its core value in addressing the limitations of single-target imaging (**Figure 2**). However, this study also has inherent limitations: the sample size remains small, and selection bias cannot be completely excluded due to the lack of a strict randomization design. Additionally, the underlying mechanism by which the bispecific probe enhances signal intensity—whether it stems from synergistic binding to dual receptors or merely additive effects—requires further verification through more in-depth molecular and cellular experiments.

Further advancing this concept, Weng et al. (65)developed LNC1015 (NOTA-RGD-RM26-03), a heterodimer targeting both αvβ3 and GRPR via conjugation of cyclic RGD and RM26. This probe was labeled with ^68^Ga and subjected to preclinical and preliminary clinical evaluations. Results demonstrated that [^68^Ga]Ga-LNC1015 exhibited favorable binding specificity and stability both *in vitro* and *in vivo*, with superior tumor uptake and retention compared to the corresponding monomeric probes. In preliminary clinical studies, the tumor uptake and TBR of both primary tumors and metastatic lesions were significantly higher than those of ^18^F-FDG PET/CT, thereby achieving higher lesion detection rates and more accurate tumor delineation (65).

The significance of this study lies in advancing the evidence for “dual-target superiority over single-target” from the early clinical exploration of BBN-RGD to next-generation probes based on RM26, while demonstrating lower hepatic uptake and more favorable pharmacokinetic profiles. However, it should be emphasized that the current evidence only supports its “preliminary clinical feasibility” rather than its status as an “established preferred option”. This is primarily due to the limited number of patients enrolled, the absence of mature outcome data, and the lack of systematic comparisons with tracers more closely aligned with breast cancer molecular subtyping, such as FES and HER2-targeted tracers. In the future, under the premise of clarifying the receptor expression profile, it is necessary to compare the incremental value of dual-target probes with single-target probes and standard imaging pathways, to determine whether they truly alter staging, biopsy strategies, or treatment decisions.

Overall, the greatest potential of GRPR-targeted imaging in breast cancer lies primarily in clinical scenarios including ER-positive/HER2-negative subtypes, low FDG avidity, invasive lobular carcinoma, predisposition to bone metastasis, and prominent molecular heterogeneity (41, 43, 47). Based on existing evidence, GRPR-targeted imaging is not a simple replacement for traditional imaging modalities, but rather serves as a bridge connecting “pathological subtyping” and “systemic molecular distribution”: it not only helps compensate for the limitations of mammography and MRI in terms of specificity or accessibility, but may also fill the blind spot of ^18^F-FDG PET in low-metabolic luminal tumors (39–41, 43, 47). However, it is crucial to objectively recognize that this field is still in the transition phase from “proof-of-concept” to “clinical evidence accumulation”. The current major bottlenecks include generally small sample sizes, insufficient stratification of breast cancer subtypes, GRPR expression heterogeneity, the impact of menstrual/endocrine status on normal breast uptake, the lack of unified quantitative thresholds and interpretation standards, and the paucity of prospective studies truly focused on clinical decision-making changes and patient outcomes as endpoints.

In our view, the most reasonable future development path for GRPR-targeted imaging should not focus on pursuing “higher SUV values” or “novel radionuclides” in isolation, but should revolve around three key questions: first, clarifying which breast cancer molecular subgroups are most suitable for GRPR imaging; second, defining the complementary boundaries between GRPR imaging and FDG, FES, HER2-targeted imaging as well as MRI; third, advancing from single-target diagnosis to dual-target stratification and theranostic validation. Only with substantial progress in these three aspects can GRPR-targeted imaging truly integrate into the precise diagnosis and treatment workflow of breast cancer, rather than remaining merely a promising research tool.

### 2.3 Lung cancer

Lung cancer remains one of the malignancies with the heaviest global disease burden, where early diagnosis and accurate staging are directly linked to treatment strategy selection and patient prognosis. Latest epidemiological data show that there were approximately 2.48 million new lung cancer cases and 1.82 million cancer-related deaths worldwide in 2022, both ranking among the leading malignancies globally; Asia accounts for approximately 63% of the world’s new cases and cancer-related deaths, indicating that this region faces particularly prominent clinical needs in lung cancer prevention, control, and precision diagnosis and treatment (66). Currently, ^18^F-FDG PET/CT remains the most commonly used molecular imaging tool for the diagnosis, staging, and efficacy evaluation of lung cancer, especially non-small cell lung cancer (NSCLC), and has been widely applied in the differentiation of benign and malignant thoracic lesions (67–69).

However, ^18^F-FDG is essentially a glucose metabolic tracer rather than a tumor-specific probe; thus, its sensitivity may decrease in slowly proliferating tumors with low metabolic activity. Furthermore, high uptake of ^18^F-FDG is often observed in infections, inflammation, and granulomatous lesions, leading to false positives and limiting its specificity in the diagnosis of complex pulmonary lesions (70, 71). Therefore, the development of novel targeted imaging agents that can more directly reflect tumor biological phenotypes and improve the ability to differentiate between tumors and inflammation holds clear clinical value.

Against this backdrop, the GRPR has emerged as a promising target due to its aberrant expression in lung cancer. Mattei et al. (72)performed IHC analysis on a large cohort of lung cancer tissues, and the results showed that the expression rates of GRPR were approximately 62.5% in NSCLC and 52.6% in small cell lung cancer (SCLC), with no significant overall difference between the two subtypes. Notably, strong GRPR expression was more likely to be observed in advanced cases, particularly in lung adenocarcinoma. The significance of this study lies in being one of the earlier studies to histologically confirm the widespread presence of GRPR across different lung cancer subtypes, suggesting that GRPR may not only serve as a molecular imaging target but also be associated with disease progression (72, 73).

Furthermore, Moody et al. (74)identified the underlying mechanism: GRPR activation induces the secretion of neuregulin-1 (NRG-1) and transactivates HER4 in a reactive oxygen species (ROS)-dependent manner, ultimately promoting NSCLC cell proliferation via the MAPK pathway rather than the PI3K/AKT pathway. This finding reinforces the functional significance of GRPR in lung cancer biology and provides a mechanistic basis for its role as a theranostic target (74–76). However, these two categories of studies have obvious limitations: the former was primarily based on archived tissue samples, which are insufficient to illustrate *in vivo* imaging feasibility, receptor accessibility, and spatiotemporal heterogeneity of receptor expression; the latter focused mainly on *in vitro* and mechanistic research, failing to directly demonstrate the net clinical benefit of GRPR blocking or tracing in clinical diagnosis and treatment.

At the current stage, the value of GRPR in lung cancer should be understood as a potential target with sufficient biological rationality but inadequate clinical validation.

Given the significant molecular heterogeneity of lung cancer, reliance on a single receptor target is often insufficient to cover all lesions, thereby highlighting the importance of bispecific targeting strategies. Integrin αvβ3 is involved in angiogenesis, invasion, and metastasis of lung cancer, while GRPR primarily reflects tumor cell phenotypes, and their combination holds strong theoretical complementarity (77–80). Liu et al. (81)developed a bispecific heterodimer probe, Glu-c(RGDyK)-bombesin, which targets both integrin αvβ3 and GRPR, and conjugated it with HYNIC to chelate ^99m^Tc, resulting in the synthesis of ^99m^Tc-RGD-BBN. Systematic evaluations of cellular binding, *in vivo* distribution, planar γ imaging, and small-animal SPECT/CT were conducted in Lewis lung cancer subcutaneous xenografts, lung metastases, and dual inflammation-tumor models.

Results demonstrated that ^99m^Tc-RGD-BBN achieved dual-specific targeting both *in vitro* and *in vivo*, with significantly lower uptake in inflammatory foci compared to ^18^F-FDG, thereby exhibiting higher specificity in differentiating tumors from inflammation. Additionally, the probe enabled clear visualization of primary lung cancer lesions and lung metastases (81). The key significance of this study lies in providing a feasible approach to address the clinical challenge of “tumor-inflammation differentiation” in the lung, while verifying that the bispecific design combining GRPR and αvβ3 can partially overcome the issue of heterogeneous single-target expression.

Nevertheless, this study remains a typical preclinical proof-of-concept: the Lewis lung cancer model differs from human lung cancer in terms of receptor profile, tumor microenvironment, and inflammatory background; SPECT also lags behind PET in spatial resolution and quantitative capability. Furthermore, the probe showed high uptake in organs with high physiological GRPR expression, such as the pancreas, indicating that potential background interference requires further optimization. In the future, it is more worthwhile to translate such bispecific constructs to PET radionuclide platforms and conduct head-to-head comparisons with more mature tracers (e.g., ^18^F-FDG, ^68^Ga-FAPI) in clinical cohorts, to validate their real incremental value in solitary pulmonary nodules and complex inflammatory lesions.

Beyond the GRPR/αvβ3 dual-target strategy, the combined targeting of GRPR and folate receptor α (FRα) also holds considerable appeal. FRα, a membrane protein associated with high-affinity folate transport, is upregulated in subsets of NSCLC, particularly lung adenocarcinoma, thereby emerging as a potential diagnostic and therapeutic target (82–85). Meanwhile, gold nanoparticles and PAMAM dendrimers have been widely used in the construction of tumor nanotheranostic platforms in recent years, attributed to their high modifiability, diverse imaging modalities, and excellent loading capacity (86–88). Wang et al. (89)constructed a ^177^Lu-labeled composite nanoplatform, ^177^Lu@DenAuNMs-bombesin-folate, using generation 4 PAMAM dendrimers as the scaffold, encapsulating gold nanomaterials within the dendrimers, and conjugating bombesin (targeting GRPR), folic acid (targeting FR), and DOTA chelators. Studies demonstrated that this system exhibited high radiochemical purity and favorable stability, showing significant and specific cellular uptake *in vitro*, as well as excellent tumor retention and imaging contrast in tumor-bearing mice (89).

The core value of this work lies in its shift beyond “pure imaging” to an integrated theranostic design, which attempts to combine dual receptor targeting, SPECT imaging, radioligand therapy, and the optical properties of nanomaterials into a single platform. Nevertheless, such nanosystems are still far from clinical translation, with several prominent limitations: first, FRα expression in lung cancer exhibits histological and interindividual heterogeneity, meaning this strategy is not suitable for all lung cancer patients; second, the reticuloendothelial system (RES) uptake, long-term *in vivo* retention, metabolic clearance pathways, and potential immunotoxicity of nanomaterials require more comprehensive evaluation; third, the multi-component composite system is significantly more complex than small-molecule peptide probes in terms of GMP manufacturing, consistency control, and regulatory approval.

In our view, the greater significance of this study lies in “demonstrating the potential of platform-based design” rather than indicating that such systems are ready for near-term clinical application. To enhance translational prospects in the future, priority should be given to simplifying the structure, clarifying the dominant targeting mechanism, optimizing pharmacokinetic profiles, and validating efficacy and safety in orthotopic lung cancer and metastatic models that more closely mimic clinical settings.

Overall, the advantages of GRPR-targeted strategies in lung cancer are primarily reflected in two aspects: first, as a receptor-type target, GRPR is theoretically closer to “tumor-specific” imaging compared to ^18^F-FDG, holding promise for improving the ability to differentiate malignant lesions from infectious/inflammatory lesions; second, GRPR can be easily combined with other targets such as αvβ3 and FRα to construct bispecific or multifunctional probes, thereby better addressing the significant molecular heterogeneity of lung cancer (72, 81, 89). However, this field currently faces common shortcomings. First, clinical evidence is significantly insufficient; most studies remain in the *in vitro* or animal model stage, lacking large-sample, prospective studies oriented toward real clinical problems. Second, receptor expression heterogeneity has not been fully quantified; differences in GRPR expression across different lung cancer subtypes, stages, treatment statuses, and even among different lesions in the same patient may affect the diagnostic stability of probes. Third, many early studies still adopted bombesin-based agonist configurations, while recent research on GRPR radiopharmaceuticals generally prefers antagonists, as the latter typically exhibit more favorable pharmacokinetic profiles and safety; this suggests that future probe optimization in lung cancer should also draw more on the “antagonist-first” design principle. Fourth, most existing studies use “imaging feasibility” as the endpoint, but what truly determines clinical value is whether it can alter staging judgments, reduce unnecessary biopsies, improve the accuracy of differentiating inflammation from tumors, or optimize radiotherapy and systemic therapy decisions.

Overall, GRPR-targeted molecular imaging provides a biologically rational new direction for the precision diagnosis and treatment of lung cancer. The work of Mattei et al. (72)and Moody et al. (74)has laid the histological and mechanistic foundation for GRPR as a potential theranostic target in lung cancer; the study of ^99m^Tc-RGD-BBN by Liu et al. (81)demonstrated the potential of the bispecific targeting strategy in improving the ability to differentiate lung cancer from inflammation; the ^177^Lu@DenAuNMs-bombesin-folate platform constructed by Wang et al. (89)further expanded the application boundary of GRPR in integrated nanotheranostics. In our view, the true breakthrough of GRPR in lung cancer does not lie in replacing ^18^F-FDG, but in serving the scenarios where ^18^F-FDG is insufficient—such as pulmonary lesions with complex inflammatory backgrounds, tumors with low metabolic activity but high receptor expression, and heterogeneous lesions requiring multi-target combined characterization.

Future research should focus on three key areas: first, conducting head-to-head clinical studies based on pathological gold standards to clarify the incremental value of GRPR probes relative to ^18^F-FDG; second, strengthening patient stratification to identify the histological subtypes and molecular subgroups most suitable for GRPR imaging; third, transitioning gradually from multi-component systems that are “conceptually complex but translationally difficult” to clinical candidate probes that are “structurally simpler, mechanistically clearer, and process-controllable”. Only by making substantial progress in these key areas can GRPR-targeted imaging truly evolve from a promising experimental tool to a clinical application in the precision medicine of lung cancer.

### 2.4 Gastrointestinal stromal tumor

GISTs are the most common mesenchymal tumors of the gastrointestinal tract, generally thought to originate from interstitial cells of Cajal (ICCs) or their precursor cells (90). At the molecular level, GISTs are not a single disease but a heterogeneous group of tumors defined by distinct driver events: KIT-mutant and PDGFRA-mutant subtypes account for the majority, with the remainder including succinate dehydrogenase (SDH)-deficient, neurofibromatosis type 1 (NF1)-associated, BRAF-mutant, and a small number of other rare subtypes (91, 92). This molecular stratification not only determines tumor biological behavior but also directly influences sensitivity to targeted therapy and imaging manifestations. Clinically, most GISTs present as localized lesions at initial diagnosis; however, recurrence, peritoneal dissemination, and distant metastasis are not uncommon during the disease course, particularly posing significant clinical challenges in high-risk patients and those with drug resistance. Although CT and MRI remain the fundamental imaging tools for initial diagnosis and follow-up, their ability to detect small intramural gastric lesions, early peritoneal implants, and residual viable lesions after treatment is still limited. While ^18^F-FDG PET/CT facilitates staging and efficacy evaluation, it essentially reflects glucose metabolism rather than GIST-specific biology, and its specificity is thus affected by inflammation, treatment response patterns, and tumor metabolic heterogeneity. Therefore, the development of non-invasive imaging strategies that more closely align with GIST molecular phenotypes holds clear clinical necessity (93, 94).

Against this backdrop, the GRPR has emerged as a promising new target. Early receptor autoradiography studies have shown a high frequency of GRPR expression in primary and metastatic GISTs, laying a foundation for subsequent targeted imaging and theranostic research (95). Subsequently, *in vitro* studies further confirmed that GRPR-related peptides can achieve significant and specific binding in GIST cell lines, and their receptor binding and internalization characteristics remain generally stable after conjugation with radionuclides (96). The significance of these findings lies in demonstrating that GRPR is not merely a “histologically detectable” marker, but also feasible for translation into *in vivo* molecular imaging probes. However, the evidence level of studies at this stage remains primarily at the ex vivo tissue and cellular levels, which is insufficient to address key questions such as *in vivo* receptor accessibility, interlesional heterogeneity, and the impact of treatment status on imaging. Therefore, at the current stage, GRPR should be regarded as a potential target with “sufficient biological basis but initial clinical validation” in GISTs, rather than an established routine imaging standard.

The head-to-head study of [^68^Ga]Ga-NOTA-RM26 PET/CT conducted by Wang et al. (97)represents a crucial milestone in the translation of GRPR imaging in GIST. Employing a prospective design, this study compared the performance of [^68^Ga]Ga-NOTA-RM26 with ^18^F-FDG PET/CT in GIST evaluation. Results demonstrated that GRPR-targeted PET/CT exhibited significantly higher lesion detection rate and uptake intensity than ^18^F-FDG, with tracer uptake positively correlated with tumor GRPR expression (97). This suggests that [^68^Ga]Ga-NOTA-RM26 is not a mere duplication of metabolic imaging, but rather provides more biologically meaningful information related to the target in GIST. Its clinical value is mainly reflected in three aspects: first, improving the sensitivity for detecting primary and small-volume lesions; second, providing additional information for differentiating GIST from some benign mesenchymal lesions; third, establishing a basis for patient selection for subsequent GRPR-targeted radioligand therapy.

It should be noted that this study primarily focused on pathologically accessible lesions, with a small sample size, and insufficient coverage of patients with advanced disease, extensive metastasis, or long-term tyrosine kinase inhibitor (TKI) exposure. In the future, a more worthwhile direction is to conduct stratified analysis by molecular subtype, TKI exposure status, and lesion location in a larger cohort, and link these analyses to treatment outcomes, to clarify whether RM26 imaging is more suitable for “diagnostic stratification” or “treatment selection” (97).

Parallel to the development of RM26, another major research line is NeoB/NeoBOMB1. [^68^Ga]Ga-NeoBOMB1 (now commonly referred to as ^68^Ga-NeoB) is a DOTA-conjugated GRPR antagonist, and preclinical studies have demonstrated its high affinity, favorable *in vivo* stability, and high tumor uptake (98). In the MITIGATE-NeoBOMB1 Phase I/IIa study, researchers first verified its safety, pharmacokinetics, and preliminary tumor-targeting ability in patients with advanced, post-TKI-treated GISTs. The results indicated that the tracer was generally well-tolerated and could exhibit tumor uptake in patients (99). The significance of this study lies not in definitively proving its superiority over existing standards at a single stroke, but in completing the critical transition from preclinical research to initial human feasibility. Its limitations are also clear: the number of enrolled cases was small, with the primary focus on safety and dosimetry rather than genuine verification of diagnostic efficacy; meanwhile, the high heterogeneity of lesions in the context of advanced disease and multi-line treatment means the results are more appropriately interpreted as “feasible” rather than “confirmed effective”. In the authors’ view, the true value of NeoB series studies lies in providing an entry point for subsequent therapeutic pairing (e.g., ^177^Lu-NeoB), rather than merely adding a new diagnostic imaging agent (100).

Subsequently, Gruber et al. (101)further evaluated the tumor-targeting performance of ^68^Ga-NeoB PET/CT in patients with advanced GISTs. The results showed that ^68^Ga-NeoB uptake exhibited significant heterogeneity: positive lesions were observed in approximately two-thirds of the patients, accounting for less than half of all lesions, while the tracer was also able to detect some CT-occult lesions (101). This finding is critical, as it suggests that the value of GRPR imaging in GIST does not lie in “universally high uptake in all patients”, but rather in identifying a specific subgroup of patients with imageable and intervenable targets. Furthermore, ^68^Ga-NeoB tended to accumulate in morphologically active non-necrotic regions, indicating that it reflects “receptor-accessible active tumor tissue” rather than the total tumor volume. The limitation of this study also stems from this characteristic: when the tracer is mainly localized to active regions, the imaging range may be smaller than the overall lesion scope shown by CT/MRI, thereby leading to an underestimation of the total tumor burden. Future improvement directions should include: combining with CT/MRI for overall lesion volume correction, conducting more refined comparisons with pathological necrosis and proliferation markers, and exploring more appropriate interpretation metrics—such as TBR—instead of relying solely on SUVmax (100).

Among the subtypes of GIST, wild-type GIST (wtGIST), particularly succinate dehydrogenase (SDH)-deficient wtGIST, represents one of the most challenging subsets in precision diagnosis and treatment of GIST. Existing studies have shown that approximately 15% of adult GISTs and the majority of pediatric GISTs lack KIT/PDGFRA driver events, a considerable proportion of which are SDH-deficient; such tumors are more commonly observed in young patients, tend to arise primarily in the stomach, and have a chronic but protracted clinical course with limited benefit from conventional tyrosine kinase inhibitors (TKIs) (102, 103). Hulse et al. (104)evaluated ^68^Ga-NeoB PET/CT in 12 patients with metastatic SDH-deficient wtGIST, and the results showed that even within the same molecular subtype, uptake remained highly heterogeneous both interlesionally and interpatiently, with distinct visible uptake lesions observed in approximately two-thirds of the patients (104).

The significance of this study is straightforward: it is the first to suggest at the human cohort level that a subset of SDH-deficient wtGIST patients can be identified and potentially selected for ^177^Lu-NeoB therapy via GRPR imaging. Meanwhile, the study also revealed a critical limitation: the lesion scope visualized by ^68^Ga-NeoB was generally smaller than that shown by CT/MRI or ^18^F-FDG PET, indicating that GRPR imaging is more akin to a “target distribution map” rather than a comprehensive surrogate for total tumor burden. Therefore, in wtGIST, the most reasonable positioning of GRPR PET is for patient selection and target confirmation, rather than independently undertaking the tasks of efficacy evaluation or total tumor burden measurement (105).

Overall, existing evidence demonstrates a clear clinical translational logic for GRPR-targeted molecular imaging in GISTs: first, GRPR exhibits imageable expression in a substantial proportion of GISTs, providing a molecular basis for receptor-specific imaging; second, the two major probe classes (RM26 and NeoB) have advanced clinical application in two distinct directions—”optimization of diagnostic performance” and “feasibility of therapeutic pairing”; third, wtGIST, particularly succinate dehydrogenase (SDH)-deficient wtGIST, may represent the most promising indication for future development, as this patient population has limited options for standard treatment and thus has an urgent need for novel targeted diagnostic and therapeutic approaches. Meanwhile, this field still faces several common bottlenecks: first, the sample size of most studies remains small, and the majority are single-center early-stage explorations; second, GRPR expression exhibits significant intra-lesional, inter-lesional, and post-treatment heterogeneity; third, GRPR PET reflects “target-expressing active tumor tissue” rather than total tumor volume, and thus cannot simply replace CT or MRI; fourth, there is a lack of prospective studies focusing on endpoints such as changes in patient management, improvement in progression-free survival (PFS)/overall survival (OS), or prediction of treatment benefit.

In our view, the most practical breakthrough for GRPR in GISTs lies not in replacing CT, MRI, or ^18^F-FDG, but in serving as a stratification tool: identifying patients who are more likely to benefit from GRPR-targeted RLT, particularly in TKI-resistant and wtGIST settings. Future research should prioritize three key areas: establishing unified imaging interpretation criteria and positive threshold standards; conducting prospective studies stratified by molecular subtype and treatment status; and directly linking GRPR imaging to therapeutic trials such as ^177^Lu-NeoB to validate its true predictive value. Only by achieving the transition from “imageable” to “treatment-guiding” can GRPR-targeted imaging truly integrate into the precision diagnosis and treatment workflow of GISTs (106).

### 2.5 Other GRPR-expressing tumors with emerging theranostic relevance

#### 2.5.1 Gliomas

Beyond the currently best-studied tumor entities, gliomas represent another malignancy in which GRPR may have diagnostic and theranostic relevance. Early immunohistochemical studies demonstrated GRPR positivity in human glioma specimens, whereas non-neoplastic glial cells were negative, supporting the biologic rationale for receptor-targeted approaches in this setting. This concept has already advanced to human imaging: in adult glioma patients, ^68^Ga-NOTA-Aca-BBN ^(^7–14) PET/CT successfully identified MRI-detected lesions, and in pediatric optic pathway glioma, GRPR-targeted PET further demonstrated excellent tumor-to-background contrast and feasibility for PET/MRI-guided surgical planning in selected cases (107–109).

However, the therapeutic translation of GRPR-targeted strategies in brain tumors faces additional barriers that are less prominent in extracranial malignancies. The blood-brain barrier (BBB) and blood-brain tumor barrier substantially restrict drug delivery, and infiltrative tumor margins may remain insufficiently exposed even when parts of the lesion enhance on conventional imaging. Potential solutions include the development of smaller and more stable ligands, locoregional administration, convection-enhanced delivery, and transient BBB-opening approaches such as hyperosmolar methods or MR-guided focused ultrasound (110); In parallel, recent preclinical work in glioblastoma models suggests that antagonist-based GRPR radioligands such as ^177^Lu-RM2 may achieve more favorable *in vivo* tumor uptake and tumor-to-normal tissue ratios than agonist comparators (111).

#### 2.5.2 Ovarian cancer

Ovarian cancer may also deserve brief discussion as a potential GRPR-associated malignancy. Earlier molecular studies reported GRPR mRNA expression in a substantial proportion of human ovarian cancer specimens, and specific bombesin binding was confirmed in a subset of tested tumors, indicating that GRPR is present and potentially targetable in at least some ovarian cancer subgroups. In addition, preclinical therapeutic studies using bombesin/GRPR-targeted cytotoxic analogs showed inhibitory activity in experimental ovarian cancer models, supporting the biologic plausibility of receptor-guided therapeutic strategies in this disease. Nevertheless, compared with prostate cancer, breast cancer, and glioma, the theranostic evidence base in ovarian cancer remains much less mature, and dedicated clinical GRPR imaging or radionuclide therapy data are still limited (85, 112, 113).

## 3 Innovative development of radionuclide targeted therapy based on GRPR

Although cancer treatment has gradually advanced from traditional local therapy to the era of molecular stratification, it still faces inherent challenges overall, including inadequate control of recurrence and metastasis, significant therapeutic heterogeneity, and unavoidable toxic side effects. Surgery, radiotherapy, and chemotherapy remain core therapeutic modalities for primary and locoregional lesions, but their long-term control over disseminated lesions and micrometastases is limited. While targeted therapy and immunotherapy have improved the prognosis of some patients, they are generally constrained by factors such as primary or acquired resistance, intra- and inter-tumor heterogeneity, and marked variability in patient benefits (114, 115). Against this backdrop, RLT, which integrates molecular recognition specificity with ionizing radiation cytotoxicity, is regarded as one of the most promising systemic precision treatment strategies to date. Its core principle is to use small molecules, peptides, or antibodies as targeting carriers to deliver therapeutic radionuclides to tumor cells or the tumor microenvironment, thereby maximizing the tumor absorbed dose while minimizing irradiation to normal tissues (116). For GRPR-positive tumors, this strategy is particularly appealing, as GRPR possesses the characteristics of expression across multiple cancer types and cell surface accessibility, making it highly suitable for integrated “diagnosis-selection-treatment” development (117)(**Table 2**).

**Table 2:** Representative GRPR-targeted therapeutic radioligands and optimization strategies.

Platform/category	Main radionuclide(s)	Key optimization strategy	Main strengths	Main limitations and current status
RM2/RM26-based antagonist platforms	^177^Lu, ^68^Ga	Shift from agonist-based constructs to antagonist scaffolds to improve *in vivo* performance; antagonists bind GRPR without receptor activation and often provide more favorable tumor uptake and tumor-to-background contrast despite lower internalization	High receptor affinity and one of the most established backbones for GRPR theranostics	Physiological uptake in organs such as the pancreas remains a concern; therapeutic clinical evidence is still limited
NeoB-based theranostic platforms	^68^Ga/^177^Lu	Integrated diagnosis-selection-therapy workflow	Well suited for patient selection, dosimetry, and matched theranostic development	Still lacks large prospective studies demonstrating clear clinical benefit
Next-generation ^177^Lu ligands	^177^Lu	Linker redesign and pharmacokinetic engineering to improve tumor retention and tumor-to-kidney ratios	Represents a transition from simple receptor targeting toward optimization of the overall therapeutic index	Evidence remains largely preclinical; generalizability across tumor types is uncertain
NEP inhibition-assisted strategies	Mainly ^177^Lu-labeled peptide ligands	Neprilysin inhibition to reduce enzymatic degradation and prolong *in vivo* stability	Enhances metabolic stability, tumor uptake, and tumor retention	Optimal timing, safety profile, and impact on normal-organ dosimetry remain to be defined
^161^Tb-based therapeutic systems	^161^Tb	Use of favorable electron emission properties to improve killing of micrometastatic and small-volume disease	Theoretically advantageous for small lesions and micro-metastases; a potential refinement beyond ^177^Lu	Clinical evidence remains limited; production, dosimetry, and accessibility require further development
^212^Pb/alpha-emitter platforms	^212^Pb	Incorporation of high-LET alpha therapy to enhance tumor-cell kill	May overcome some efficacy limitations of beta emitters in resistant or difficult-to-treat disease	Toxicity boundaries, organ protection, and optimal indications remain unclear; currently at an early stage
Accessible technetium/rhenium platforms	^99m^Tc, ^188^Re	Focus on clinical accessibility, lower cost, and practical implementation of individualized dosimetry	Compatible with routine nuclear medicine infrastructure and potentially favorable for broader translation	Quantitative performance, image resolution, or therapeutic efficacy may be inferior to more advanced PET or therapeutic radionuclide platforms
Dual-target or hybrid constructs	GRPR/PSMA, GRPR/integrin αvβ3	Dual-target design to address lesion underdetection caused by receptor heterogeneity	May improve lesion coverage and applicability in biologically heterogeneous tumors	Increased molecular complexity may complicate pharmacokinetics, manufacturing, and clinical translation

One of the primary considerations surrounding GRPR-targeted radioligand therapy (GRPR-RLT) is how to enhance *in vivo* metabolic stability and optimize tumor/kidney dose distribution while maintaining high receptor affinity. Kanellopoulos et al. (118)screened four ^177^Lu-labeled candidate molecules based on two GRPR antagonist scaffolds, [NMe-Gly^11^]RM26 and DB15, and conducted systematic preclinical comparisons. The results demonstrated that all these compounds could be efficiently labeled and retain high GRPR affinity; among them, AU-SAR-M1 exhibited the optimal comprehensive pharmacokinetic profiles after the introduction of a neprilysin (NEP) inhibition strategy, characterized by high and sustained tumor uptake, relatively low renal retention, and the most favorable tumor-to-kidney AUC ratio.

The significant implication of this study is that the optimization of GRPR-RLT should not merely focus on “identifying molecules that can bind to the receptor”, but should further advance to the level of “improving the therapeutic index”. In particular, the NEP inhibition-based “metabolic protection” strategy provides a practical approach for peptide radiopharmaceuticals to overcome rapid *in vivo* degradation. However, its limitations are evident: the existing evidence is mainly derived from PC-3 mouse models and cannot be directly extrapolated to different tumor types or humans; additionally, the optimal administration timing, safety margins, and impact of NEP inhibition on normal organ dosimetry in clinical settings still require prospective verification.

In our view, the true value of this work lies in shifting the research focus of GRPR-RLT from “pure ligand development” to “pharmacokinetic engineering optimization”, which provides instructive insights for all subsequent peptide-based radiotherapeutic platforms (119).

A closely related key direction is to directly enhance the serum stability of ligands through chemical modification. Felber et al. (120)developed [^177^Lu]Lu-AMTG as a representative of this approach. By modifying the RM2 scaffold with α-methyltryptophan, AMTG exhibited higher metabolic stability in preclinical studies; subsequently, the team conducted the first human serum stability evaluation in 4 patients with metastatic castration-resistant prostate cancer (mCRPC). Results showed that approximately 82% ± 6% of the intact parent compound remained 1 hour after injection, which was significantly higher than the reported level of the previous control GRPR ligand [^68^Ga]Ga-RM2 (120). This finding indicates that AMTG is not only “more stable” *in vitro* but also truly demonstrates stronger resistance to enzymatic degradation in humans. Its significance lies in the fact that a higher intact serum rate typically translates to more stable targeted delivery, more sustained tumor exposure, and greater potential for therapeutic development.

Caution is warranted, however: the study had an extremely small sample size, and the primary endpoint was pharmacokinetics rather than efficacy or toxicity, thus it is still insufficient to directly demonstrate that [^177^Lu]Lu-AMTG is superior to previous ligands in terms of clinical outcomes. In the future, it is more worthwhile to systematically link serum stability, tumor uptake, absorbed dose, and efficacy indicators to establish a complete evidence chain of “structural modification—pharmacokinetic improvement—dosimetric benefit—efficacy enhancement” (120).

At the radionuclide level, ^161^Tb is considered a potential key upgrade over ^177^Lu. The two nuclides share similar half-lives and average beta-minus (β^-^) energies; however, ^161^Tb additionally emits low-energy electrons, thereby enhancing local energy deposition at the microscopic scale. Theoretically, this makes it particularly suitable for the treatment of microscopic lesions and single-cell residual disease (121, 122). Holzleitner et al. (123)compared the preclinical performance of RM2 and AMTG labeled with ^161^Tb and ^177^Lu, respectively. The results showed that all four radioligands retained high affinity, with most radioactivity localized to the cell membrane rather than undergoing significant internalization—consistent with the typical behavior of GRPR antagonists. Among them, AMTG derivatives, regardless of the radionuclide used, exhibited superior *in vivo* stability and tumor retention compared to RM2, with [^161^Tb]Tb-AMTG achieving the highest tumor uptake.

This study holds two key implications: first, it reaffirms that “ligand stability” may have an impact on therapeutic performance comparable to that of the radionuclide itself; second, it suggests that even in non-internalizing antagonist systems, ^161^Tb may still offer biological advantages over ^177^Lu. This provides a valuable correction to the traditional notion that “low-energy electrons must be close to the cell nucleus to be effective”. However, its limitations are evident: current conclusions are primarily derived from dosimetry and mouse distribution studies, lacking sufficient comparisons of long-term efficacy and organ toxicity. Future research should further focus on three aspects: the lesion sizes in which the therapeutic gain of ^161^Tb is manifested, whether it increases delayed injury to normal tissues such as the kidneys, and its real clinical cost-effectiveness relative to ^177^Lu (123).

While ^161^Tb represents a refined upgrade of the β therapy platform, targeted alpha therapy (TAT) embodies an alternative therapeutic modality with enhanced cytotoxicity. Compared with β-emitters, alpha particles exhibit higher linear energy transfer (LET) and shorter tissue range, theoretically enabling irreversible DNA double-strand breaks (DSBs) with fewer particle tracks while minimizing bystander irradiation to distant normal tissues (124–126). Within this framework, Saidi et al. (127)developed a GRPR-targeted alpha therapeutic ligand, [^212^Pb]Pb-DOTAM-GRPR1, and conducted systematic evaluations in PC-3 prostate cancer xenograft models. Results demonstrated that this molecule possessed nanomolar affinity and favorable *in vivo* stability, achieving high tumor uptake and prolonged retention; although the kidneys served as the primary dose-limiting organ, no significant toxicity was observed at the highest tested dose. Notably, this therapy extended the median survival of tumor-bearing mice from 9 weeks in the control group to 19 weeks, with some animals achieving long-term tumor-free survival (127).

This study provides compelling proof-of-concept for GRPR-TAT, indicating that even non-internalizing receptor-targeting systems can achieve significant antitumor efficacy through high-LET radiation. Nevertheless, the clinical translation of TAT faces prominent challenges, including daughter nuclide redistribution, precise assessment of renal toxicity, tolerance to repeated treatment courses, and the complexity of radionuclide production and supply chains. In our view, the value of [^212^Pb]Pb-DOTAM-GRPR1 lies not only in its “potent efficacy” but also in its implication that the GRPR platform may not be limited to β therapy in the future; instead, radionuclide strategies with different LET values can be selected based on tumor burden and spatial scale (127).

Beyond ligands and radionuclides themselves, theranostic pairing remains a pivotal link for RLT to truly enter the era of personalized medicine. Kanellopoulos et al. (128)evaluated the therapeutic potential of two RM26-derived antagonist peptides labeled with ^188^Re, and further explored whether their ^99m^Tc-labeled counterparts could serve as tools for SPECT diagnosis and dosimetric prediction. The results suggested that the ^188^Re and ^99m^Tc versions exhibited favorable consistency in *in vivo* distribution trends, while the therapeutic probe showed even higher actual uptake in tumors—indicating that pre-therapeutic dosimetric estimation using ^99m^Tc is practically feasible but may be conservative (128). The significance of this approach lies in the fact that the ^99m^Tc/^188^Re pair belongs to a highly accessible generator-based radionuclide system; if a stable and reproducible theranostic correlation can be established, it would facilitate the translation of GRPR-RLT from highly specialized centers to more widespread clinical application.

However, its limitations are evident: current evidence remains at the preclinical level, and GRPR antagonist peptides may exhibit the issue that diagnostic and therapeutic versions are not fully equivalent under different chelator systems. In the future, a more reasonable direction for improvement is to systematically compare the consistency between imaging dosimetry and actual therapeutic absorbed dose under the premise of unifying ligand scaffolds and labeling conditions, and to explore generator-based theranostic platforms that are more suitable for routine clinical translation (129).

Overall, research on GRPR-targeted RLT is undergoing a clear evolutionary process: early work focused on validating GRPR as a viable therapeutic target; subsequently, the research focus shifted to enhancing *in vivo* stability, optimizing tumor/kidney distribution, screening for superior therapeutic radionuclides, and constructing implementable theranostic pairing systems (118, 120–128). Based on current evidence, AMTG represents the direction of “improving the therapeutic index through ligand engineering”, ^161^Tb represents the direction of “enhancing microscopic cytotoxicity through the physical properties of radionuclides”, ^212^Pb represents the direction of “breaking the upper limit of efficacy through high-LET alpha particles”, and ^99m^Tc/^188^Re represents the direction of “improving clinical accessibility and the feasibility of individualized dosimetry”. The biggest bottleneck at this stage lies not in the lack of candidate molecules, but in the lack of evidence that can truly address clinical questions: which patients are most suitable for GRPR-RLT, what imaging thresholds can be used for enrollment screening, which types of lesions are more suitable for beta-emitters or alpha-emitters, how to balance efficacy with toxicity to normal organs such as the kidneys and pancreas, and how to standardize the integration of NEP inhibition or other metabolic protection strategies into the treatment workflow.

In our view, the most important task for GRPR-RLT in the next stage is not to continue simply expanding the range of “more radionuclides and more ligands”, but to establish a systematic development framework centered on patient selection, dosimetry, organ protection, and clinical outcomes. Only when GRPR imaging can stably predict treatment benefits, and GRPR-RLT can demonstrate a net clinical benefit over existing therapies in specific patient populations, can this direction truly evolve from a “promising radiopharmaceutical development platform” to a mature precision therapeutic strategy (117).

## 4 Challenges and prospects

Currently, the core challenge facing GRPR-targeted theranostics is no longer “whether receptor-specific imaging can be achieved”, but rather “whether treatment decisions can be guided by stable and reproducible biological signals”. On the one hand, GRPR expression exhibits distinct dependence on tumor type, molecular subtype, and disease stage: it holds greater application potential in estrogen receptor-positive breast cancer, whereas in advanced or castration-resistant prostate cancer (CRPC), GRPR expression may decrease, thereby compromising the benefits of targeted therapy. On the other hand, previous systemic therapy may also alter GRPR expression and radioligand uptake, suggesting that a single baseline imaging result cannot be fully equated with sustained treatment sensitivity. Therefore, establishing patient selection criteria based on tumor type, molecular subtype, prior treatment history, and dynamic imaging results is the primary prerequisite for the standardized application of GRPR-targeted therapy (130–133).

**Figure 3:**
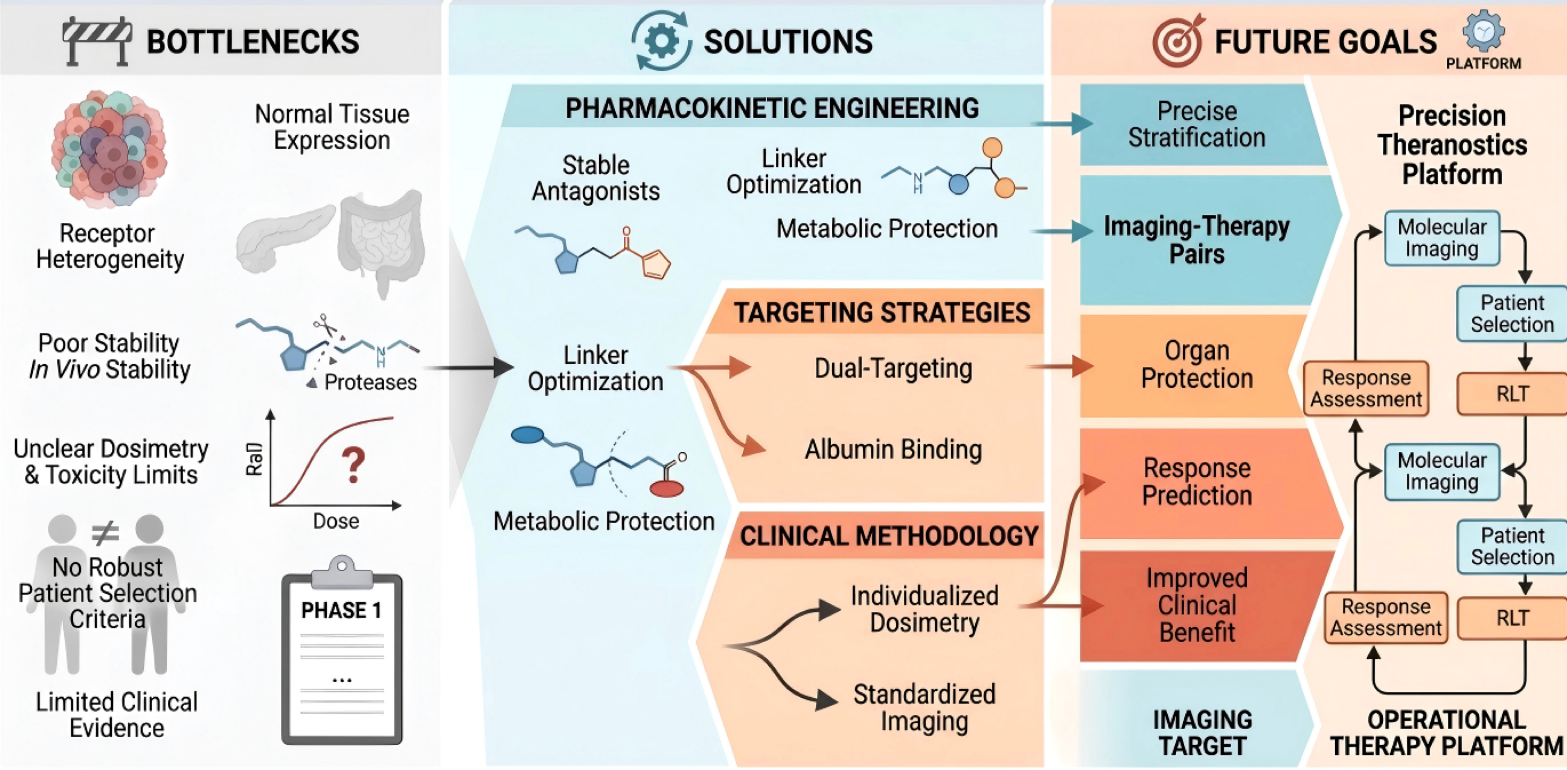
Major translational bottlenecks and future directions of GRPR-targeted theranostics. The image outlines the principal challenges hindering the clinical translation of GRPR-targeted theranostics, including limited ligand stability, heterogeneous receptor expression, suboptimal target coverage, and insufficient standardization of radionuclide selection and therapeutic assessment. It also highlights major future directions, such as radioligand optimization, dual-target design, expansion to radionuclides with distinct radiobiological properties, and construction of an integrated workflow combining GRPR PET, individualized dosimetry, and treatment response evaluation.

Second, the key to GRPR-targeted radioligand therapy (GRPR-RLT) lies not in merely enhancing receptor affinity, but in improving the overall therapeutic index (118). Existing studies have generally established that antagonists are superior to agonists, and this difference is not merely empirical but also has a plausible molecular and pharmacologic basis (131, 134). As a class A G protein-coupled receptor, GRPR undergoes conformational activation upon agonist binding, which promotes downstream signaling and is typically followed by receptor activation and internalization (131). By contrast, antagonists occupy the receptor binding pocket without triggering receptor activation and are thought to preferentially stabilize inactive receptor conformations. For radiotheranostic applications, this distinction is highly relevant, because the *in vivo* performance of a radioligand depends not only on internalization, but also on the number of accessible receptor binding states, duration of receptor occupancy, metabolic stability, and clearance from normal tissues. Accordingly, although agonists may exhibit stronger signaling-associated internalization, antagonists often achieve higher tumor uptake and more favorable tumor-to-background ratios *in vivo* while avoiding unwanted physiologic stimulation (131, 134). However, peptide ligands still commonly face challenges such as *in vivo* metabolic instability, short circulation time, and limited normal organ dose. Early human dosimetric studies have shown that while ^177^Lu-RM2 exhibits considerable tumor uptake, the pancreas serves as a key limiting organ. In recent years, through “pharmacokinetic engineering” strategies—including linker reconstruction, improved protease stability, and NEP inhibition—the new generation of GRPR antagonists has demonstrated superior tumor retention and tumor-to-organ ratios in preclinical studies (118, 134, 135). This suggests that the optimization direction of GRPR-RLT should shift further from “identifying bindable molecules” to “systematically improving the overall therapeutic index” (**Figure 3**).

Thirdly, a key bottleneck limiting the clinical translation of GRPR-targeted therapy lies in the failure of the evidence hierarchy to advance from “feasibility verification” to “demonstration of clinical benefit”. At the current stage, GRPR PET has demonstrated favorable diagnostic value in diseases such as prostate cancer, breast cancer, and GISTs; however, evidence for the therapeutic arm remains dominated by preclinical studies and early-phase clinical trials, with a paucity of prospective studies capable of addressing critical clinical questions. These include: what imaging thresholds can serve as treatment enrollment criteria, which dosimetric parameters are most closely associated with efficacy and toxicity, and whether GRPR-targeted therapy can truly improve patient management and clinical outcomes compared to existing therapeutic pathways. For a multi-cancer target such as GRPR, future research must place greater emphasis on individualized dosimetry, standardized interpretation, and the design of efficacy endpoints, rather than merely focusing on “lesion visualization” or “acceptable short-term safety” (116, 133, 136, 137).

Looking ahead, the development of GRPR-targeted theranostics is likely to advance along four key lines: first, building on high-stability antagonists, further improving *in vivo* exposure and therapeutic index through strategies such as linker optimization, albumin binding, or metabolic protection; second, developing bispecific configurations such as GRPR/PSMA and GRPR/αvβ3 to mitigate missed detection and undertreatment caused by receptor heterogeneity; third, optimizing therapeutic radionuclide selection based on lesion scale and drug-resistant backgrounds, further exploring radionuclides with distinct radiobiological characteristics (e.g., and) beyond; fourth, integrating GRPR PET, individualized dosimetry, and treatment response assessment into a closed-loop workflow, promoting the transformation of GRPR from an “imaging target” to a precise therapeutic platform that can truly guide treatment. In particular, for α-emitting systems such as, individualized dosimetry should not rely solely on macroscopic organ-level estimates derived from parent-radionuclide biodistribution. Although the elementally matched theranostic pair provides an attractive basis for quantitative SPECT imaging and patient-specific treatment planning, accurate dose estimation remains challenging because acts as an *in vivo* generator of α-emitting daughters, and daughter nuclide redistribution may lead to a spatial mismatch between the distribution of the parent radioligand and the actual sites of α-particle energy deposition. In particular, partial release of after decay has been reported, raising concern that off-target migration of daughter nuclides may increase dose to normal tissues and reduce the accuracy of conventional dosimetric assumptions. Therefore, future GRPR-targeted α-therapy studies should incorporate not only pre-therapy surrogate imaging, but also correction models for daughter migration, chelator stability, and lesion-level microdosimetry, so that individualized dosimetry can better reflect the true biologically relevant dose delivered to both tumors and organs at risk (124, 126, 127). Only by synchronously advancing probe optimization, clinical validation, standardized interpretation, and biologically informed dosimetry can GRPR-targeted theranostics be translated from a promising radiopharmaceutical platform into a mature precision oncology strategy (116, 130, 131, 134, 137).
